# Risk factors for surgical difficulty in interval appendectomy for perforated appendicitis with abscess in children

**DOI:** 10.1007/s00383-025-06157-w

**Published:** 2025-08-19

**Authors:** Keisuke Suzuki, Chizuka Seki, Tsubasa Goshima, Mina Yoshida, Yuta Nakashima, Yuki Shiko, Maiko Osawa, Yohei Kawasaki, Yujiro Tanaka

**Affiliations:** 1 Department of Pediatric Surgery, Saitama Medical University Hospital, 38 Morohongo, Moroyama-cho, Iruma-gun, Saitama, 350-0495 Japan; 2 Department of Biostatistics, Graduate School of Medicine, Saitama Medical University, Saitama, Japan

**Keywords:** Perforated appendicitis, Appendiceal abscess, Interval appendectomy, Difficulty

## Abstract

**Purpose:**

To predict surgical difficulty during interval appendectomy for perforated appendicitis with abscess.

**Methods:**

The records of children diagnosed with appendiceal abscess who underwent interval appendectomy from 2012 to 2024 were reviewed. The clinical data associated with difficult surgeries (operative time > 2 h or addition of trocars) and uncomplicated surgeries were compared.

**Results:**

Among the 61 children who underwent interval appendectomy, 42 had uncomplicated surgery, whereas 19 children had difficult surgery. Children who underwent difficult surgery were older (11.5 ± 3 vs. 8.8 ± 3 years, *p* = 0.001), taller (*p* = 0.009), and weighed more (*p* = 0.011) compared to those who had uncomplicated surgery. In addition, difficult surgery was associated with larger abscesses (*p* = 0.003) and longer initial hospital stay (*p* = 0.046). Multivariate analysis identified older age (OR = 1.36; 95% CI = 1.08–1.8; *p* = 0.017), longer abscess diameter (OR = 1.36; 95% CI = 0.96–2.02; *p* = 0.097), and longer initial hospital stay (OR = 1.03; 95% CI = 1–1.07; *p* = 0.097) as possible risk factors for difficult surgery. According to ROC analysis, the cut-off values of age, abscess diameter, and length of hospital stay for predicting complicated appendectomy were 10.4 years, 5.8 cm, and 16.5 days, respectively.

**Conclusion:**

Older age, larger abscesses, and longer hospital stay for non-operative treatment may contribute to increased surgical difficulty during interval appendectomy.

## Introduction

Acute appendicitis is one of the most common conditions in children that require urgent abdominal surgery. About 20–74% cases of acute appendicitis are complicated with appendiceal perforation [1, 2], and the perforated appendix is often surrounded by omentum that forms a localized walled-off abscess [1]. Emergent appendectomy is considered a high-risk approach for appendicitis with abscess due to the presence of inflamed tissues and adhesion around the appendix [2]. Therefore, such cases are increasingly being treated through a non-surgical approach consisting of intravenous antibiotics and abscess drainage, followed by interval appendectomy [3–7].

Several studies have shown that compared to emergent appendectomy, interval appendectomy is a better option for treating appendicitis with abscess due to shorter operative duration [5] and lower rates of overall complications [6] such as wound infection and postoperative intra-abdominal abscess [3]. Nevertheless, for some patients at our institute, interval appendectomy was challenging to perform even several months after non-operative treatment due to the appearance of severe peri-appendiceal adhesions. Increased surgical difficulty often requires longer operative time or additional trocars for laparoscopic surgery and may increase the rate of complications like injury to the surrounding tissues and bleeding. In fact, several reports on interval appendectomy include cases with operative duration longer than 2 or 3 h [8, 9]. However, the risk factors of surgical difficulty in interval appendectomy for perforated appendicitis with abscess have not been identified so far.

The aim of this study was to identify the factors influencing the surgical difficulty in performing interval appendectomy after non-operative treatment for perforated appendicitis with abscess, and enable preoperative prediction of the operative difficulty. To this end, we compared the clinical data of the children who had difficult surgery and those requiring uncomplicated procedures during interval appendectomy for appendiceal abscess.

## Methods

### Participants

This study was approved by the Saitama Medical University Hospital’s Medical Ethics Committee (approval number, 2024–122), and was conducted in accordance with the hospital’s ethical policy and the principles of the Declaration of Helsinki 1964. We retrospectively reviewed the medical records of pediatric patients (aged 15 years or younger) who were diagnosed with perforated appendicitis with abscess, and received intravenous antibiotics with or without abscess drainage, followed by elective interval appendectomy between July 2012 and December 2024. Patients who required unplanned appendectomy due to failure of non-operative treatment, the recurrence of appendicitis, or unexpected complications such as small bowel obstruction were excluded.

### Diagnosis

The diagnosis of acute appendicitis and appendiceal abscess was established by pediatric surgeons. When acute appendicitis was suspected based on the symptoms, physical examination, and blood tests, the formation of abscess, defined as an encapsulated fluid collection, was confirmed by ultrasonography (US) or computed tomography (CT) scans.

### Therapeutic strategy of acute appendicitis

Emergent surgery is generally recommended at our institute for cases of acute appendicitis with peritonitis. As an exception, interval appendectomy after non-operative treatment is indicated for perforated appendicitis with abscess when the patient’s overall physical condition is stable and the peritonitis is localized.

### Non-operative treatment

The abscess was drained via the transrectal or transabdominal route when it could be punctured safely, and the following broad-spectrum antibiotics with efficacy against anaerobic bacteria were administered intravenously: (1) sulbactam/ampicillin (SBPT/ABPC) with ceftazidime (CAZ), and (2) carbapenems like imipenem/cilastatin (IPM/CS), meropenem (MEPM) and doripenem (DRPM) with or without metronidazole (MNZ). The antibiotics were selected based on the bacterial species that were frequently isolated from pus cultures at our institute, including *E. coli*, *Enterococcus* spp., *Pseudomonas aeruginosa*, and *Bacteroides fragilis*. The regimen was changed to second-line antibiotics if the white blood cell (WBC) count or C-reactive protein (CRP) level remained high. Emergent appendectomy was performed when the abscess did not shrink and inflammatory markers did not improve despite abscess puncture and change in antibiotic regime. Non-operative treatment was terminated when the WBC count was normalized, CRP levels dropped below 1 mg/dl, and the abscess was undetectable by US. Oral antibiotics were given after discharge for 1 to 2 weeks in case of delayed normalization of WBC count and CRP during hospitalization.

### Interval appendectomy

Interval appendectomy was performed approximately 3 months after completion of the non-operative treatment, according to the previous reports indicating an interval of 2 to 4 months [3, 5, 10]. Appendectomy was conducted via single-incision laparoscopy-assisted surgery through umbilical incision [11]. After the surrounding adhesion was dissected, the appendix was exteriorized through the umbilical incision. The mesoappendix and the appendix were resected under direct vision. In cases where it was difficult to bring the appendix outside the abdomen, all procedures were performed laparoscopically. Furthermore, a skin incision was made for an extra trocar in case the surgeon deemed that operation through a single incision was difficult. All procedures were performed by experienced pediatric surgeons. The patients were discharged when they could tolerate oral feedings and gained adequate pain control.

### Study design

The operative time one standard deviation above the mean for all appendectomies at our institute— including that of emergent surgery for uncomplicated appendicitis— was approximately 120 to 125 min (data not shown). Therefore, a duration of 2 h was set as a threshold for defining a difficult appendectomy. We also identified technical difficulty by reviewing operative records and videos. Accordingly, the patients were divided into two groups according to the surgical difficulty at interval appendectomy : (1) difficult surgery group— patients with operative duration longer than 2 h or patients who required an extra trocar other than the umbilicus, and (2) easy surgery group. The following variables were compared between the two groups: age, gender, height, body weight, body mass index (BMI), duration of symptoms, body temperature, WBC counts, CRP levels at diagnosis, maximum diameter of abscess, presence of appendicolith, detectability of appendix on imaging, abscess drainage, change in intravenous antibiotics to second-line antibiotics, whether oral antibiotics was given at discharge, and interval between discharge from non-postoperative treatment and appendectomy.

### Statistical analysis

All data processing and statistical analyses were performed using R software (version 4.4.2). A *p* value of < 0.05 was considered statistically significant. Continuous variables have been expressed as mean ± SD, and categorical variables as frequencies and percentages. Continuous variables were compared using Student’s *t* test and categorical variables were analyzed with Chi-square test or Fisher’s exact test. Univariate and forward stepwise multivariate logistic regression analyses were conducted to identify risk factors for difficult surgery at the time of interval appendectomy (*p* value threshold of 0.10). Odds ratios (ORs) with 95% confidence intervals (CIs) and *p* values were calculated. The receiver operating characteristic (ROC) curve was plotted for the risk factors identified by multivariate analysis, and the optimal cut-off values for the incidence of difficult surgery were determined using the Youden Index. The sensitivity and specificity at the determined cut-off values were also calculated.

## Results

### Outcomes of interval appendectomy and surgical difficulty

**Fig. 1:**
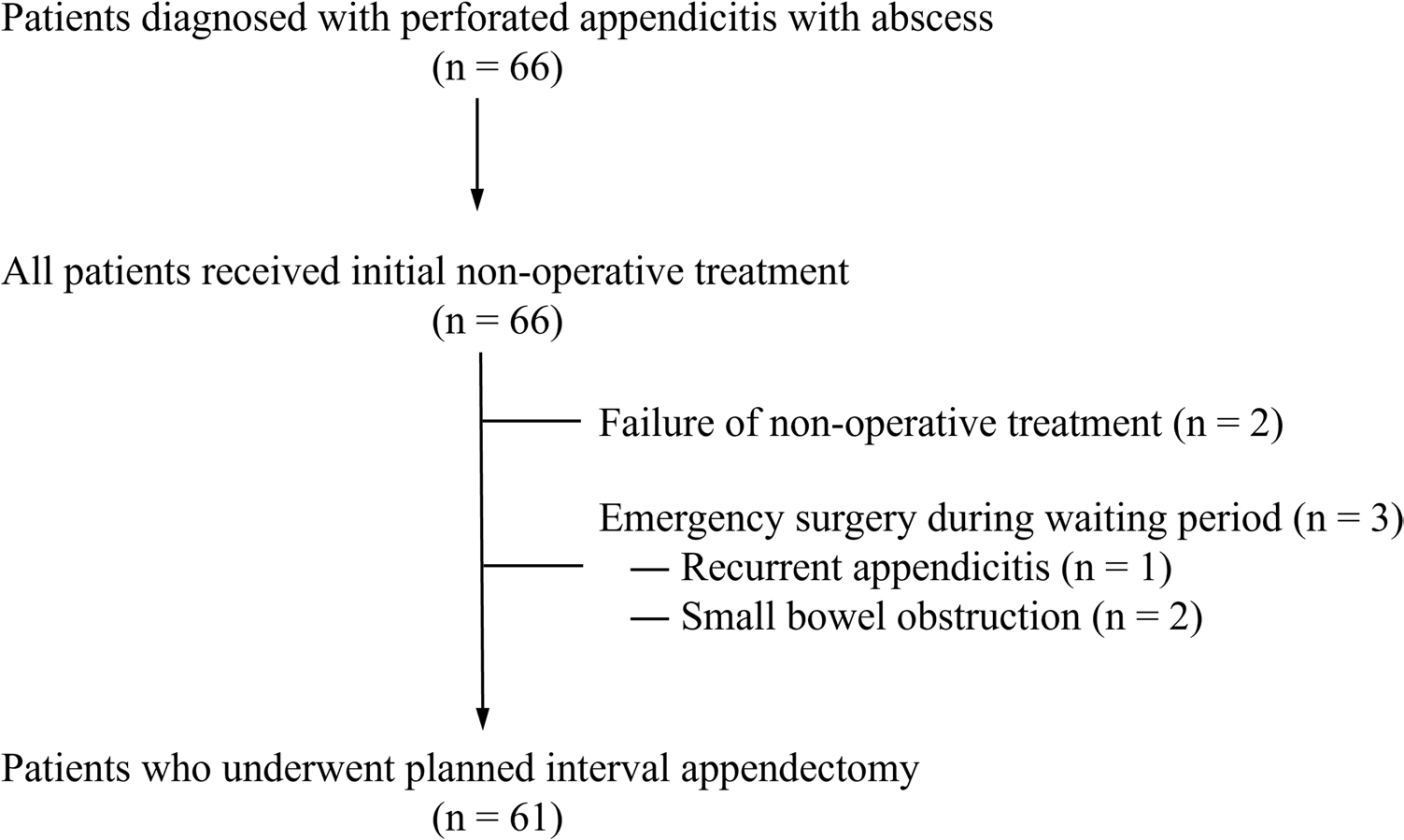
Clinical course of patients diagnosed with perforated appendicitis with abscess

Sixty-six children were diagnosed with perforated appendicitis with abscess between 2012 and 2024 at our institute (Fig. 1). Initially, all patients underwent non-operative treatment. Two patients did not respond to the non-operative treatment due to abscess and the recurrence of inflammation shortly after abscess shrinkage, and therefore underwent emergent appendectomy. Three patients required emergent surgery during the waiting period— one due to recurrent appendicitis and the others due to small bowel obstruction. Surgical difficulty was evaluated in the remaining 61 patients who underwent planned interval appendectomy, and the outcomes have been summarized in Table 1. Nineteen patients required difficult surgery, and forty-two patients had easy surgery at the time of interval appendectomy. The operative duration was 154.4 ± 44.2 min in the difficult surgery group and 68.1 ± 22.8 min in the easy surgery group. Extra trocars other than the umbilicus were inserted in 15 patients (79%) with difficult surgery due to challenges in dissecting the adhesion. The postoperative length of stay was significantly longer in the difficult surgery group (4.4 ± 2.3 days vs. 2.5 ± 1.0 days, *p* < 0.001). However, the incidence of complications was not significantly different between the two groups (15.7 vs. 4.8%, *p* = 0.17). Two patients in the easy surgery group and one in the difficult surgery group developed infection at the surgical site. In addition, two patients in the difficult surgery group experienced injuries in the seromuscular layer of the small bowel, which were repaired intraoperatively.

**Table 1:** Operative outcomes of interval appendectomy

	Easy surgery (*n* = 42)	Difficult surgery (*n* = 19)	*p* value
Operative duration (min)	68.1 ± 22.8	154.4 ± 44.2	< 0.001
Addition of extra trocars	–	15 (78.9%)	
Postoperative length of stay (days)	2.5 ± 1.0	4.4 ± 2.3	< 0.001
Complications	2 (4.8%)	3 (15.7%)	0.17

### Patient demographics and clinical characteristics at the time of diagnosis

The demographic and clinical characteristics of patients in the two groups have been summarized in Table 2. Patients with difficult surgery were older than those with easy surgery (11.5 ± 3 years vs. 8.8 ± 3 years, *p* = 0.001). In addition, the height (145.2 ± 16 cm vs. 131.4 ± 19.3 cm, *p* = 0.009) and body weight (39.5 ± 10.8 kg vs. 30.8 ± 12.6 kg, *p* = 0.011) were significantly higher in the difficult surgery group. BMI was also higher in the difficult surgery group, albeit without statistical significance (18.5 ± 3.3 vs. 17 ± 3, *p* = 0.071).

**Table 2:** Patient demographics and clinical characteristics at the time of diagnosis

	Easy surgery (*n* = 42)	Difficult surgery (*n* = 19)	*p* value
Gender (M/F)	24: 18	10: 9	0.960
Age (years)	8.8 ± 3.0	11.5 ± 3.0	**0.001**
Body height (cm)	131.4 ± 19.3	145.2 ± 16.0	**0.009**
Body weight (kg)	30.8 ± 12.6	39.5 ± 10.8	**0.011**
BMI	17.0 ± 3.0	18.5 ± 3.3	0.071
Duration of symptoms (days)	4.6 ± 3.0	4.5 ± 3.5	0.867
Body temperature ≧ 38.0℃	24 (57.1%)	13(68.4%)	0.581
WBC count ≧ 15,000/μl	27(64.3%)	11(57.9%)	0.848
CRP ≧ 10 mg/dl	24 (57.1%)	9 (47.4%)	0.666
Diameter of abscess (cm)	4.1 ± 1.5	5.6 ± 2.3	**0.003**
Appendicolith	27 (64.3%)	16 (84.2%)	0.202
Detectability of appendix	27 (64.3%)	14 (73.7%)	0.667

Values in bold indicate statistical significance (*p* < 0.05)

The duration of symptoms at the time of appendiceal abscess diagnosis was similar in both groups (4.5 ± 3.5 days vs. 4.6 ± 3 days, *p* = 0.867). Likewise, the proportion of patients with WBC counts higher than 15,000/μl and CRP level > 10 mg/dl was not significantly different between the two groups. The maximum diameter of the abscess, measured on the basis of CT scan or US findings, was significantly longer in the difficult surgery group than in the easy surgery group (55.8 ± 23.3 mm vs. 40.5 ± 15.1 mm, *p* = 0.003). However, other imaging findings, such as the presence of appendicolith and detectability of the appendix, were not significantly different between the two groups.

### The clinical course of non-operative treatment and interval appendectomy

The details of non-operative treatment have been summarized in Table 3. Abscess drainage was performed for six patients (31.6%) in the difficult surgery group and for ten patients (23.8%) in the easy surgery group (*p* = 0.746). There were no differences in the drainage route between the two groups. Thirteen patients in the difficult surgery group (68.4%) and eighteen patients in the easy surgery group (42.9%) switched to second-line antibiotics from intravenous antibiotics (*p* = 0.116), and the proportion of patients who required oral antibiotics after discharge also did not show any significant difference between the two groups (12 cases (63.2%) vs. 26 cases (61.9%), *p* = 1.000). The duration of hospital stay for non-operative treatment was significantly longer in the difficult surgery group (13.7 ± 5.1 days vs. 11.5 ± 3.3 days, *p* = 0.046), while the interval between non-operative treatment and appendectomy was similar in both groups (109.4 ± 31.7 days vs. 109.8 ± 28.5 days, *p* = 0.962).

**Table 3:** Details of non-operative management

	Easy surgery (*n* = 42)	Difficult surgery (*n* = 19)	*p* value
1st-line antibiotics			0.886
SBT/ABPC + CAZ	24 (57.1%)	12 (63.1)	
Carbapenem antibiotics	16 (38.1%)	6 (31.6%)	
others	2 (4.8%)	1 (5.3%)	
Change to second-line antibiotics	18 (42.9%)	13 (68.4%)	0.116
Drainage	10 (23.8%)	6 (31.6%)	0.746
Transabdominal^*1^	7 (16.7%)	4 (21.1%)	
Transrectal^*1^	4 (9.5%)	2 (10.5%)	
Oral antibiotics after discharge	26 (61.9%)	12 (63.2%)	1.000
Length of hospital stay (days)	11.5 ± 3.3	13.7 ± 5.1	**0.046**
Interval between discharge and appendectomy (days)	109.8 ± 28.5	109.4 ± 31.7	0.962

Value in bold indicates statistical significance (*p* < 0.05)

^*1^Some cases were counted more than once due to overlapping drainage procedures

### Univariate and multivariate analyses

As shown in Table 4, the age, height, body weight, and diameter of the abscess were significantly correlated with surgical difficulty. In the multivariate analysis (Table 5), older age was the only independent factor affecting surgical difficulty (OR = 1.36; 95% CI = 1.08–1.80; *p* = 0.017). The length of hospital stay for non-operative treatment (OR = 1.16; 95% CI = 0.99–1.39; *p* = 0.084) and the diameter of abscess (OR = 1.36; 95% CI = 0.96–2.02; *p* = 0.097) may also be associated with an increased risk of surgical difficulty, although neither reached statistical significance.

**Table 4:** Univariate analysis of risk factors for difficult surgery

Variables	Unadjusted odds ratio (95% CI)		*p* value
Gender	0.83	(0.28–2.50)	0.743
Age	1.39	(1.13–1.77)	**0.004**
Body height	1.05	(1.01–1.09)	**0.014**
Body weight	1.06	(1.01–1.12)	**0.016**
Duration of symptoms	0.98	(0.81–1.17)	0.865
Body temperature ≧ 38.0℃	0.62	(0.19–1.88)	0.406
WBC ≧ 15,000 /μl	1.31	(0.42–3.97)	0.634
CRP ≧ 10 mg/dl	1.48	(0.50–4.48)	0.479
Diameter of abscess	1.56	(1.15–2.24)	**0.008**
Appendicolith	2.96	(0.82–14.22)	0.124
Detectability of appendix	1.56	(0.49–5.58)	0.471
Drainage	1.48	(0.43–4.87)	0.524
Change to second-line antibiotics	2.89	(0.95–9.62)	0.069
Oral antibiotics	1.05	(0.35–3.55)	0.925
Length of hospital stay	1.14	(1.00–1.32)	0.054
Interval between discharge and appendectomy	1.00	(0.98–1.02)	0.961

Values in bold indicate statistical significance (*p* < 0.05)

**Table 5:** Multivariate analysis of risk factors for difficult surgery

Variables	Adjusted odds ratio (95% CI)		*p* value
Age	1.36	(1.08–1.80)	**0.017**
Length of hospital stay	1.16	(0.99–1.39)	0.084
Diameter of abscess	1.36	(0.96–2.02)	0.097

Value in bold indicates statistical significance (*p* < 0.05)

The optimal cut-off values of these variables were determined by ROC analysis, and were as follows: 10.4 years for age (sensitivity 68.4%; specificity 73.8%; AUC = 0.711), 5.8 cm for the diameter of abscess (sensitivity 42.1%; specificity 90.5%; AUC = 0.663), and 16.5 days for the length of hospital stay for non-operative treatment (sensitivity 31.6%; specificity 92.9%; AUC = 0.622). Based on these values, we set practical cut-off values as follows: age ≧ 11 years, diameter of abscess ≧ 6 cm, and length of hospital stay ≧ 17 days. A positivity for any one or more of the three variables could predict difficult surgery with a sensitivity of 84.2%, specificity of 66.7%, positive predictive value of 53.3%, and negative predictive value of 90.3%.

## Discussion

This study is the first study to evaluate causes of difficult surgery in pediatric patients with appendiceal abscess at the time of interval appendectomy. Our findings suggest that age, height, body weight, abscess diameter, and the length of hospital stay for non-operative management may influence surgical difficulty at interval appendectomy for perforated appendicitis with abscess. In addition, older age was identified as an independent risk factor for difficult surgery in the multivariate analysis, while larger abscess and longer hospital stay for non-operative management were also suggested as potential risk factors. Hosokawa et al. had previously reported that pre‑operative CT findings such as intra‑abdominal fat density around the appendix and retrocecal or retro‑ascending colon appendix were risk factors for prolonged operative time for interval appendectomy. However, no cases with abscess were included in that study [9]. Furthermore, Iqbal et al. could not identify any risk factors for complications of interval appendectomy in patients with perforated appendicitis [12].

The impact of age on the risk of surgical difficulty can be attributed to several factors. First, abdominal incision and closure generally require more time in older children due to their thicker abdominal wall. Second, the distance between umbilicus and cecum increases, and the flexibility of the abdominal wall decreases as children grow [13], which in turn increases the difficulty in exteriorizing the appendix through the umbilicus. Third, older children might have severe adhesions, which contribute to increased surgical difficulty. According to operative records and surgical videos, patients who underwent difficult surgeries exhibited wider and more rigid adhesions. There are several possible explanations for the association between age and adhesion. The greater omentum, which traverses the peritoneal cavity and sequesters areas of inflammation and injury, develops by the age of 11 years [14], which corresponded to the cut-off value for difficult surgery identified in the present study. The greater omentum gradually grows after birth and covers a wider area of the intestine and accumulates more fat tissue with age [15]. Therefore, the mature greater omentum in older children may cover the perforated appendix more thoroughly, resulting in more rigid and wider adhesion. In addition, age-related changes in the coagulation cascade and the fibrinolytic system may cause more severe intra-abdominal adhesion in older children. A large amount of fibrinogen is released from the damaged peritoneum during infection or abdominal surgery and is converted to fibrin by the coagulation cascade. The fibrinolytic system, which lyses fibrin deposits in the normal state, can be compromised due to infection or abdominal surgery. The resulting accumulation of fibrin in the abdominal cavity eventually causes adhesion [16]. The coagulation cascade and the fibrinolytic system develop throughout childhood, and the concentration of key components differs with age [17, 18], which may influence intra-abdominal adhesion after perforated appendicitis with abscess.

Larger abscesses and longer hospital stay for non-operative treatment also appeared to be risk factors for difficult surgery. Although these factors did not reach statistical significance, they reflect the severity of local inflammation around the appendix, and a more rigid and wider adhesion can be left as the abscess shrinks, thereby increasing the difficulty of appendectomy. On the other hand, other inflammatory indicators such as high body temperature and elevated WBC counts and CRP levels were not associated with surgical difficulty. It is possible that these indicators do not necessarily represent the local inflammation or the severity of the abscess. The predictive value of abscess diameter and length of hospital stay will have to be validated in further studies with larger cohorts.

It was tricky to determine the appropriate timing of interval appendectomy as all procedures were scheduled according to a uniform protocol, i.e., 3 months after completion of non-operative treatment. However, within the observed variation of 109.6 ± 29.2 days— primarily due to patient convenience or surgical slot availability— the interval until appendectomy did not appear to affect surgical difficulty. Therefore, a longer interval might not decrease surgical difficulty. On the contrary, it may increase the risk of recurrent appendicitis during the waiting period, as recurrence has been reported to occur at an average of 3 months after non-operative treatment for complicated acute appendicitis [19].

Based on the results of ROC analysis, patients who are older than 11 years, have abscesses larger than 6 cm, or spend longer than 17 days in the hospital for non-operative management may be at a higher risk of difficult appendectomy. Surgeons can refer to this prediction model when scheduling interval appendectomy, and in case a patient is positive for any one or more of the above factors (age ≧ 11 years, diameter of abscess ≧ 6 cm, length of hospital stay ≧ 17 days), should consider the probability of surgical difficulty. Although the specificity (66.7%) and positive predictive value (53.3%) of this prediction model are not very high, it has remarkably high sensitivity (84.2%) and negative predictive value (90.3%). Therefore, in case a patient is negative for this predictive model and has a low risk of surgical difficulty, the interval appendectomy can be performed with an uncomplicated procedure. Furthermore, based on the results of the predictive model, the surgeon can inform the patients and their parents about the risk of prolonged operative time or additional trocar placement when obtaining informed consent.

There are several limitations in our study that ought to be considered. First, the study was retrospective and included a small number of patients. Second, as surgical difficulty is difficult to evaluate objectively, we used operative duration longer than 2 h and addition of trocars as the substitute criteria. The operative records and videos of the patients with difficult surgery indicated severe adhesion. In addition, the choice of intravenous antibiotics or use of oral antibiotics after discharge was somewhat decided at the discretion of the attending doctor, although these variations did not appear to influence the surgical difficulty. Further studies with a more unified protocol and criteria are required to validate our results.

In conclusion, older age, larger abscesses, and longer hospital stay for non-operative treatment can be risk factors for difficult surgery at interval appendectomy. Surgeons should consider the possibility of difficult surgery in patients who present any of the above risk factors and prepare accordingly.

## Data Availability

No datasets were generated or analyzed during the current study.
